# Circulating B-Lymphocytes as Potential Biomarkers of Tuberculosis Infection Activity

**DOI:** 10.1371/journal.pone.0106796

**Published:** 2014-09-05

**Authors:** Ismail Sebina, Irene A. Biraro, Hazel M. Dockrell, Alison M. Elliott, Stephen Cose

**Affiliations:** 1 Co-infection Studies Programme, Medical Research Council/Uganda Virus Research Institute Uganda Research Unit on AIDS, Entebbe, Uganda; 2 Department of Internal Medicine, College of Health Sciences, Makerere University Kampala, Uganda, Kampala, Uganda; 3 Department of Immunology and Infection, London School of Hygiene & Tropical Medicine, London, United Kingdom; 4 Department of Clinical Research, London School of Hygiene & Tropical Medicine, Keppel Street, London, United Kingdom; University of Cape Town, South Africa

## Abstract

Accurate biomarkers of *Mycobacterium tuberculosis* infection activity would significantly improve early diagnosis, treatment and management of *M. tuberculosis* infection. We hypothesised that circulating B-lymphocytes may be useful biomarkers of tuberculosis (TB) infection status in highly TB-endemic settings. *Ex-vivo* and *in-vitro* mycobacteria-specific B-cell ELISPOT assays were used to examine the plasmablast (PB) and memory B-cell (MBC) responses in the peripheral blood of adult, healthy, community controls (n = 151) and of active TB patients (n = 48) living in Uganda. Frequencies of mycobacteria-specific PBs were markedly higher in active TB patients compared to healthy controls, and, conversely, MBCs were markedly higher in the healthy controls compared to active TB patients. In addition, the community controls with evidence of latent TB infection had higher peripheral blood PB and MBC responses than those without evidence of TB infection. These data demonstrate that peripheral blood B-cell responses are differentially modulated during latent and active *M. tuberculosis* infection, and suggest that the PB to MBC ratio may be a useful biomarker of TB infection activity.

## Introduction

Until recently, tuberculosis (TB) was defined as a two-stage infection - active or latent. In contrast, current thinking suggests that TB may induce a broad spectrum of immune responses, dependent on the *Mycobacterium tuberculosis* load and/or replication activity within infected individuals, so that the interplay of mycobacterial replication and host immune response results in disease, recovery from active infection, or a sub-clinical latent state [1]–[4]. Understanding the interplay between *M. tuberculosis* and host immune responses within this clinical spectrum remains a critical research goal [5], [6]. In addition, identification of biomarkers that can accurately predict the clinical status of *M. tuberculosis* infected individuals, and identify those who are at the highest risk of progressing from sub-clinical latent infections to clinically manifested active disease, also remains a fundamental challenge [5]–[10]. Novel transcriptomic approaches have improved our knowledge of the immune response to, and pathogenesis of, TB, providing new insight into potential biological markers that might eventually stratify *M. tuberculosis* infected individuals [7], [11], [12]. One major break-through from such approaches indicated a relationship between a type 1-interferon inducible gene signature and disease activity as assessed by chest x-ray [13]. Interestingly, in this paper the healthy latent TB infected individuals had gene signatures that clustered with both active TB and healthy control gene signatures. The authors suggested that the healthy latent TB infected individuals with gene signatures clustering with those of active TB, might be the ones about to develop active TB, although they did not prove this hypothesis in this paper. Subsequently, other studies have reported changes in gene expression profiles of other signatures within whole blood of TB patients before and after treatment, as well as in latently infected individuals and healthy controls [11], [14], [15]. Although these approaches have generated exciting results, the cost and expertise needed to produce accurate results limit their practicability and efficiency, particularly in highly endemic and resource poor settings. Cheap, simplified, rapid and accurate alternatives to these approaches are therefore still needed.

Given the critical role of T-cells in *M. tuberculosis* immunity, approaches aiming to identify new TB biomarkers have previously been biased towards T-cells and T-cell associated molecules. However, recent reports suggesting various modulatory roles for B-cells in *M. tuberculosis*-specific immune responses [16]–[21] have revitalised interest in B-cells as possible targets for new biomarkers [14], [15]. In a previous study, we described higher frequencies of MBCs within the peripheral blood of healthy UK-donors who had previously visited or lived in highly TB-endemic areas compared to their counterparts who had neither visited nor stayed in such areas [22]. More recently, active TB patients who were successfully cured following treatment were found to present contrasting B-cell associated gene expression patterns within their blood, before and after treatment [14].

In the present study, we investigated whether the ratio of plasmablasts (PBs) to memory B-cells (MBCs) may accurately predict the clinical status of *M. tuberculosis* infected individuals living in a highly TB-endemic area. Plasmablasts are short-lived antibody secreting cells (ASCs) and are readily detected in peripheral blood during an early or on-going active infection, but they are rarely present in the circulation in the absence of antigen and, or, of viable, replicating pathogens [23]. In contrast, MBCs are long-lived and may persist throughout the lifetime of the host [23]–[27]. MBCs rapidly and specifically respond to antigenic re-stimulation, differentiating into new ASC populations [27]–[30]. Given their prolonged lifespan and rapid response, MBCs may contribute to the rapid clearance of pathogens following re-exposure throughout the host's lifetime [24], [25], although this has not been demonstrated for infection with *M. tuberculosis*. Nevertheless, the B cell response to infection with *M. tuberculosis* may be promising in predicting the immunological (and consequently clinical) status of *M. tuberculosis* infected individuals. We reasoned that the presence of antigen-specific PBs in the absence of MBCs within the peripheral circulation may reflect a current or on-going active *M. tuberculosis* infection (clinically manifested disease); that the presence of PBs in addition to MBCs may reflect a resolving active or a subclinical latent infection; and that the presence of MBCs in the absence of PBs may reflect sterile immunity following infection and thus a healthy state.

## Results

### PB and MBC responses to *M. tuberculosis* antigens

**Figure 1:**
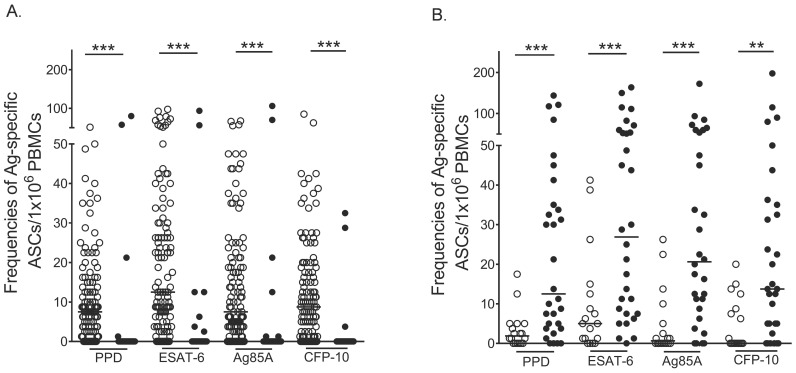
*Mycobacteria*-specific PB and MBC responses in the healthy community controls and active TB cases. Frequencies of antigen-specific PBs and MBCs were determined by 6-hour ex-vivo and 6-day in-vitro ELISPOTs respectively. Frequencies of antigen-specific PBs and MBCs are presented as antibody secreting cells per million PBMCs. Frequencies of mycobacteria-specific MBCs (open circles) and PBs (closed circles) in the healthy community controls (**A**) and active TB patients (**B**) are shown. ***, P<0.001; **, P<0.01.

Little is known about the peripheral blood B-cell responses induced by *M. tuberculosis* infection. We therefore examined these responses by employing short-term *ex-vivo* and 6-day *in-vitro* ELISPOT assays to determine *Mycobacteria*-specific PB and MBC responses, respectively, in the peripheral blood of adult, healthy, community controls (n = 151) and active TB patients (n = 48). PB and MBC responses were present and readily detected within the peripheral blood of these individuals (Figure 1). Of the 116 healthy controls tested for MBC responses, 74 had positive MBC responses to PPD, 84 to ESAT-6, 76 to CFP-10 and 74 to Ag85A (Figure 1A, open circles). Of another 35 healthy controls tested for PB response, three participants had positive PB responses to PPD, five to ESAT-6, four to CFP-10 and four to Ag85A (Figure 1A, closed circles). Conversely, of the 18 active TB patients tested for MBC responses, five had positive responses to PPD, 10 to ESAT-6, six to CFP-10 and five to Ag85A (Figure 1B). Of another 30 active TB cases, tested for PB responses, 24 had positive PB responses to PPD, 27 to ESAT-6, 25 to CFP-10 and 24 to Ag85A (Figure 1B). Healthy community controls had significantly higher frequencies of mycobacteria-specific MBCs than of PBs (Figure 1A and Figure S1A), and active TB patients had significantly higher frequencies of mycobacteria-specific PBs than of MBCs (Figure 1B and Figure S1B). We observed strong correlations amongst the detected antigen-specific PB and MBC responses, (Table 1), showing that individuals who responded to any one antigen were likely to respond to all antigens tested.

**Table 1:** Correlations among paired antigen-specific PB and MBC responses detected in the community controls and TB patients.

Association	Spearman's coefficient (r) MBCs Healthy/TB patients	P-value	Spearman's coefficient (r) PBs Healthy/TB patients	P-value
PPD and ESAT-6	0.65/0.65	<0.0001/0.0032	0.74/0.63	<0.0001/0.0002
PPD and Ag85A	0.59/0.44	<0.0001/0.0704	0.77/0.52	<0.0001/0.0035
PPD&CFP-10	0.66/0.67	<0.0001/0.0022	0.40/0.63	0.0166/0.0002
ESAT-6 and Ag85A	0.79/0.80	<0.0001/<0.0001	0.72/0.87	<0.0001/<0.0001
ESAT-6 and CFP-10	0.70/0.81	<0.0001/<0.0001	0.27/0.63	0.1163/0.0002
CFP-10 and Ag85A	0.68/0.67	<0.0001/0.0024	0.30/0.77	0.0784/<0.0001

### Differential modulation of *Mycobacteria*-specific PB and MBC responses during *M. tuberculosis* infection

**Figure 2:**
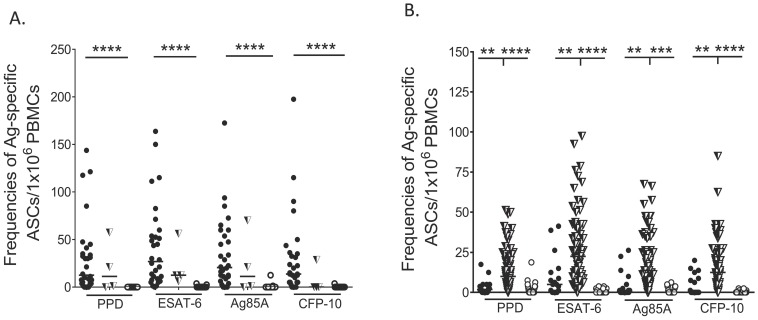
*Mycobacteria*-specific PB and MBC responses in the active TB patients and community controls. Frequencies of antigen-specific PBs and MBCs were determined by 6-hour ex-vivo and 6-day in-vitro ELISPOTs respectively. Frequencies of antigen-specific PBs and MBCs are presented as antibody secreting cells per million PBMCs. (**A**) Frequencies of mycobacteria-specific PBs in the active TB patients (filled circles), community controls responding to ESAT-6/CFP-10 (half filled triangles) and healthy community controls lacking ESAT-6 and CFP-10 responses (open circles) (**B**) Frequencies of mycobacteria-specific MBCs in the active TB patients (filled circles), community controls responding to ESAT-6/CFP-10 (half filled triangles) those without ESAT-6 and CFP-10 responses (open circles). **** P<0.0001, ***, P<0.001; **, P<0.01.

Healthy community controls were not assessed for latent TB infection using conventional assays such as the tuberculin skin test (TST) or interferon-gamma release assays (IGRAs). Thus, participants in this group may have been latently infected or truly TB-naïve. To explore this possibility, we stratified our community controls into those who had responses to ESAT-6 or CFP-10 (n = 89) and those who lacked responses to both these *M. tuberculosis* – specific antigens (n = 26) in either of the two B-cell assays. We then compared the PB and MBC responses detected in these individuals to those detected in the active TB patients. Consistent with our hypothesis, and as described above, high PB numbers were characteristic of active TB patients, although MBCs were also detected within their peripheral blood (Figure 2, filled circles). On the other hand, high MBC numbers to PPD and Ag85A were characteristic of healthy individuals with responses to ESAT-6 or CFP-10, although a few PBs were detected in some individuals (Figure 2, half filled triangles). All healthy individuals lacking responses to both ESAT-6 and CFP-10 also lacked PB responses to PPD and Ag85A, and only low numbers of MBCs were present within their peripheral blood (Figure 2, open circles). This data is consistent with the generally held definition that healthy individuals with responses to ESAT-6 and CFP-10 are latently infected, and those lacking responses to both of these antigens are uninfected [31]. For the purpose of our further analyses, by analogy with T-cell, IGRAs [31], we defined individuals who had a response to either of the two *M. tuberculosis* – specific antigens in either B-cell assay as having probable latent TB infection. Together, these data demonstrate that peripheral blood B-cell responses are differentially modulated during latent and active *M. tuberculosis* infection, and that the frequencies of *M. tuberculosis*-specific PBs and MBCs in peripheral blood may be able to accurately predict the clinical status of *M. tuberculosis* infected individuals living in highly TB-endemic settings.

### The ratio of *mycobacteria*-specific PBs to MBCs is likely to be associated with *M. tuberculosis* clinical status

**Figure 3:**
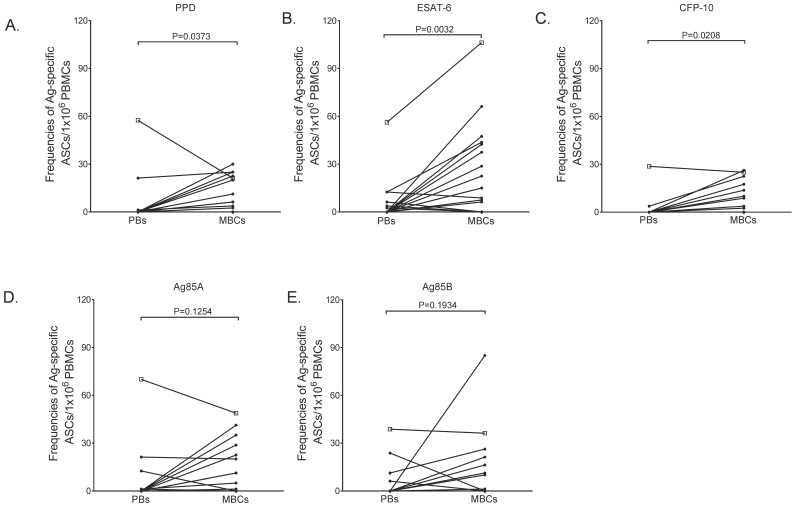
Paired *mycobacteria*-specific PB and MBC responses in healthy community controls. Matched frequencies of antigen-specific PBs and MBCs were determined by 6-hour ex-vivo and 6-day in-vitro ELISPOTs respectively. Matched frequencies of PPD- (**A**), ESAT-6- (**B**), CFP-10- (**C**), Ag85A- (**D**) and Ag85B- (**E**) specific PBs and MBCs in healthy community controls are shown. Open square across all panels represents an interesting donor who presented with a higher PB:MBC ratio on various antigens tested. Frequencies of antigen-specific PBs and MBCs are presented as antibody secreting cells per million PBMCs.

Having observed contrasting peripheral blood PB and MBC responses in the community controls and active TB patients, we next considered that the ratio of PBs to MBCs (PB:MBC) might be a more accurate approach for predicting the clinical status of *M. tuberculosis* infected individuals. Considering that we conducted most of our assays in different individuals, we could not directly assess this approach. Nevertheless, we reasoned that comparing median PB and MBC responses in active TB patients to those in the latently infected or uninfected community controls might reveal a pattern that would reflect likely PB:MBC ratios. Indeed, we observed that the median antigen-specific PB response was higher in the TB patients than in the uninfected and latently infected controls, but also higher in the latently infected than in the uninfected controls (Table 2). In contrast, the median antigen-specific MBC response was higher in the latently infected controls than in the active TB patients and uninfected controls (Table 2). This data further suggests that PBs may be associated with an active TB phenotype and MBCs with a latent state. To explore this further, we analysed data from the group of 35 healthy community controls for whom we had data on both PB and MBC responses (Figure 3). The majority of these healthy participants had more peripheral blood MBCs than PBs, presenting a low PB:MBC ratio (Figure 3). Of interest, one participant, who had not been BCG-vaccinated, presented with a ratio consistent with that of the active TB patients (Figure 3, indicated with an open square). We postulate that this individual may be more at risk of developing active TB infection than the other healthy volunteers, or may be progressing from latency towards active disease. Together, these data suggest that the ratio of *M. tuberculosis*-specific PBs to MBCs in peripheral blood may predict the clinical status of *M. tuberculosis* infected individuals, and thus could be a useful biomarker of *M. tuberculosis* infection status, particularly in individuals living in highly TB-endemic settings.

**Table 2:** Median PB and MBC responses in the community controls and active TB patients.

Antigen	Median antigen-specific cells per 10^6^ PBMCs (median PBs/median MBCs)		
	Healthy	LTBI	Active
Ag85A	0/0	11/14	20/1
CFP-10	0/0	0/13	14/0
ESAT-6	0/0	13/23	27/5
PPD	0/0	11/10	12/2

## Discussion

We have shown that peripheral blood mycobacteria-specific B-cell responses are differentially modulated during active and latent *M. tuberculosis* infection - PB responses are significantly elevated whereas MBC responses are depleted from the peripheral circulation during active TB disease. These observations may be explained by the increase in mycobacterial loads due to the presence of viable replicating mycobacteria within these TB patients. These elevated mycobacterial loads may activate circulating mycobacteria-specific MBCs, which subsequently differentiate into new PB populations, and thus contribute to the elevation of PBs detected within the peripheral blood of active TB patients. In addition, the increase in mycobacterial loads may accelerate the activation and differentiation of naive B-cells, thereby contributing to the PB pool. Plasmablasts contribute to the increase in serum levels of protective antibodies during active infection, regardless of the pathogen. Increased serum antibodies are readily detected during TB infection [32], [33], although why such antibodies are not protective remains unknown.

It should be noted that we used frozen PBMCs from active TB patients and fresh PBMCs from healthy volunteers in our experiments. There is some suggestion that the freeze/thaw cycle may affect cell viability and/or a selective loss of cell populations, notably activated cells [34], [35]. Given this potential issue, we might expect the freeze/thaw process to selectively reduce numbers of PBs, which are activated B cells; however, we could clearly identify PB populations in our thawed PBMCs from active TB patients. Thus, if the freeze/thaw process did negatively affect PB numbers, we are likely to have underestimated the high PB:MBC ratio in these patients. In addition, a recent study showed that the freeze/thaw process did not affect influenza-specific PB numbers [36], and in a more recent report, MBC responses were also largely unaffected by this process [37]. It is therefore likely that the freeze/thaw process did not significantly alter the numbers of PBs or MBCs that we report here.

Our study lacked conventional tests such as the TST or an IGRA that could independently confirm latent TB infection (LTBI) in our healthy controls. Nevertheless, our data is consistent with what we might expect from such assays: the proposed LTBI group showed responses to ESAT-6 and/or CFP10, whereas the LTBI negative group did not. In particular, the marked lack of B-cell responses to any mycobacterial antigens in those individuals with no TB-specific B-cell responses (Figure 2A and Table 2) suggests that these individuals are indeed *M. tuberculosis* naïve. Considering the relatively high prevalence of non-tuberculous mycobacteria (NTM) in sub-Saharan Africa, some of the responses to ESAT-6 and/or CFP-10 detected in the LTBI group may be attributed to previous exposure to such mycobacterial species. Although this possibility cannot be ruled out completely, it is worth noting that the prevalence of infection with such mycobacterial species in these settings is much lower compared to that of *M. tuberculosis* [38], suggesting that *M. tuberculosis* infection or exposure dominates these B-cell responses.

Our data suggests a potential role for *M. tuberculosis*-specific peripheral blood B-cell responses as biomarkers of TB infection. We propose that the *M. tuberculosis*-specific PB:MBC ratio may serve as a valuable and early diagnostic marker for both latent and active TB infections in humans. We envision that the changes in the frequency of PBs or peripheral blood PB-specific responses might be an accurate way to identify an immediate and active immune response, and thus may be a useful predictor of an individual who is currently exposed to replicating *M. tuberculosis*. If we are correct, then the raised PB:MBC ratio observed across some antigens in a single healthy community control may be particularly significant (Figure 3). Although we were unable to follow up this individual during the course of this study, our hypothesis would suggest that individuals of this phenotype may be progressing towards active, clinical disease. Clearly, further prospective studies need to be done before definitive conclusions can be made. Such studies may lead to the accurate detection of discreet stages of infection within latently infected individuals, as has been proposed [1], and subsequently to early identification of those individuals who are most likely to progress to a clinically manifested active disease. Such individuals would then be the obvious targets for directed chemoprophylaxis.

## Concluding Remarks

This study investigated whether differences in peripheral blood B-cell responses accurately reflect the clinical status of *M. tuberculosis* infected individuals. We demonstrate a differential modulation in peripheral blood PB and MBC responses during active and latent *M. tuberculosis* infection, reflected in a significant increase in the peripheral blood PB:MBC ratio during active *M. tuberculosis* infection. We conclude that the ratio of *M. tuberculosis*-specific PBs to MBCs within the peripheral blood may be a useful biomarker of TB infection activity and thus warrants further investigation. Prospective studies aiming to test the kinetics of this ratio among untreated, *M. tuberculosis* latently infected individuals who might progress towards active infection, or in newly identified active TB patients undergoing treatment, may provide better insight and validation of this concept. This may thus serve as a significant improvement to currently available methods for detecting *M. tuberculosis* infection, which cannot accurately distinguish between active and latent infections.

## Materials and Methods

### Ethics statement

The ethics committees at Makerere University, Kampala, Uganda, the Uganda Virus Research Institute (UVRI) and the Uganda National Council of Science and Technology (UNCST) approved these studies. Informed written consent was obtained from all study participants. Participants provided up to 20 ml of heparinised venous blood that was used in subsequent experiments.

### Study participants

Forty-eight (48) newly diagnosed, adult, BCG-vaccinated, HIV negative, untreated, sputum positive TB patients, 18–50 years of age, were included from a larger prospective study, at two health clinics in Kampala, Uganda. In addition, 151 adult, HIV negative, clinically healthy, community controls, 18–55 years old, were recruited through an HIV counselling and testing programme at the UVRI clinic in Entebbe, Uganda, as part of a cross-sectional study. Of the 151 healthy community controls recruited, only four had not been previously vaccinated with BCG.

### Antigens and mitogens

*Mycobacterium tuberculosis* ESAT-6 (NR-14868), CFP-10 (NR-14869), Ag85A (NR-14871) and Ag85B (NR-14870) were obtained through BEI Resources (NIAID, USA) and purified protein derivative for *in-vitro* use (PPD) from Statens Serum Institut (SSI, Denmark). Non-adsorbed tetanus toxoid (TT) was obtained from the National Institute for Biological Standards and Control (NIBSC), UK. *Phytolacca americana* Pokeweed mitogen (PWM) and *Staphylococcus aureus* Cowan (SAC) were obtained from Sigma-Aldrich, UK. CpG-oligonucleotide (ODN-2006, 5′-tcgtcgttttgtcgttttgtcgtt-3′) was obtained through Eurofins MWG/Operon, Germany.

### Study design

*In-vitro* and *ex-vivo* B-cell ELISPOT assays were used to examine the mycobacteria-specific memory B cell (MBC) and plasmabast (PB) responses, respectively, in both active TB patients and the healthy community controls. Peripheral blood mononuclear cells (PBMCs) were isolated by Ficoll Paque Plus (Amersham Pharmacia Biotech, UK) density-gradient centrifugation, washed twice in Hank's balanced salt solution (HBSS, Invitrogen, UK) and resuspended in RPMI-1640 culture media (Invitrogen, UK) containing 10% Fetal Bovine Serum (FBS), 100 U/ml penicillin, 100 mg/ml streptomycin and 2 mM L-glutamine (all from Sigma-Aldrich, UK). Purified PBMCs from the blood of the active TB patients were stored in liquid nitrogen until used; those from the healthy community controls were used fresh in the ELISPOT assays. *In-vitro* ELISPOT assays were used to determine MBC responses in 116 healthy community controls. Separately, paired *ex-vivo* and *in-vitro* ELISPOT assays were used to determine matched PB and MBC responses in PBMCs from another 35 clinically healthy participants. For the active TB patients, PBMCs from 30 participants were analyzed using the *ex-vivo* ELISPOT to determine their PB responses. PBMCs from another 18 active TB cases were analyzed using the *in-vitro* ELISPOT assay to determine their MBC response. Due to limited blood volumes and PBMC numbers obtained from the active TB patients, we were unable to conduct both assays on samples from the same patient.

### *In-vitro* ELISPOT assay

Frequencies of MBCs in peripheral blood were determined using an in-house *in-vitro* B-cell enzyme-linked immunosorbent spot (ELISPOT), previously described [22]. Briefly, purified PBMCs (1x10^6^/ml) were stimulated with a cocktail of 6 µg/ml CpG, 0.5 µg/ml PWM, 1.2 mg/ml SAC, 25 ng/ml recombinant human IL-10 (R&D systems, UK) and 50 µM β-mercaptoethanol for six days at 37°C in a humidified 5% CO_2_ incubator. Polyclonal stimulated cells (4x10^5^ cells/ml) were then transferred directly into wells of a 96-well filter plate (Millipore, MAHAS4510) that was coated with either 100 µl of PBS containing *Mycobacterium*-specific recombinant proteins (6 µg/ml PPD, 10 µg/ml ESAT-6, Ag85A, Ag85B, and CFP-10), 2 µg/ml Tetanus toxoid (TT, positive control) or with PBS alone (negative control). Plates were developed with a biotin-SP-conjugated AffiniPure fragment donkey anti-human IgG (Jackson ImmunoResearch), strepavidin- AKP (BD biosciences) and its substrate (AP-conjugate substrate kit, Biorad, USA). Developed spots were counted using an AID ELISPOT reader (AID Diagnostika, Germany) and AID ELISPOT software (version 7.0). The frequencies of antigen-specific MBCs are presented as spot counts per million stimulated PBMCs. A positive ELISPOT response was defined as the presence of two or more spots in each replicate well, with the total number of spots in antigen coated wells being at least twice that observed in the PBS-coated wells that had no more than five spots.

### *Ex-vivo* ELISPOT assay

Short term *ex-vivo* ELISPOTs were used to determine the frequencies of antigen-specific IgG secreting plasmablasts in peripheral blood of TB patients and healthy controls. Ninety-six (96) ELISPOT- well filter plates (MAHA S4510, Millipore) were coated overnight with either 100 µl of PBS containing mycobacterium-specific recombinant proteins (6 µg/ml PPD, 10 µg/ml ESAT-6, Ag85A, Ag85B, and CFP-10). Tetanus toxoid (2 µg/ml) was used as positive control while PBS-coated wells served as negative controls. Plates were washed and blocked with RPMI containing 1% BSA at 37°C for 2 hours. Purified PBMCs (2x10^5^/ml) were added into each antigen-coated well and the plates were incubated for 5 hours at 37°C in a 5% CO_2_ incubator. Plates were washed with PBS and 0.05% PBS-Tween and developed with biotin-SP-conjugated AffiniPure fragment donkey anti-human IgG (Jackson ImmunoResearch), strepavidin- AKP (BD biosciences) and its substrate (AP-conjugate substrate kit, Biorad, USA). Developed plates were air-dried and the number of resultant spots enumerated on an AID ELISPOT reader. Frequencies of antigen-specific PBs were calculated and later presented as spot forming units per million PBMCs.

### Statistical analysis

Collected data were entered into Excel and GraphPad Prism software. Statistical analysis was performed using Stata 10.1. Frequencies of PBs and MBCs were not normally distributed and therefore non-parametric statistical tests were used. Comparisons of antigen-specific PB and MBC responses between healthy community controls and TB patients were made using an unpaired Wilcoxon sign rank test. Matched PB and MBC responses in a selected set of healthy community controls were compared using a paired Wilcoxon sign rank test. Spearman's rank correlation coefficient was used to test for correlations amongst various antigen-specific PB and MBC responses. Only statistically significant differences are shown.

## Supporting Information

Figure S1 **Mycobacteria-specific PB and MBC responses in the healthy community controls and active TB cases.** Frequencies of antigen-specific PBs and MBCs were determined by 6-hour ex-vivo and 6-day in-vitro ELISPOTs respectively. Frequencies of antigen-specific PBs and MBCs are presented as antibody secreting cells per million PBMCs. Frequencies of mycobacteria-specific PBs in healthy community controls (open circles) and active TB patients (closed circles) (A) and MBCs in healthy community controls (open circles) and active TB patients (closed circles) (B) are shown. ****, P<0.0001 ***, P<0.001; **, P<0.01, *, P<0.05.
(TIF)
