# Supplementary figures and images for: Circulating B-Lymphocytes as Potential Biomarkers of Tuberculosis Infection Activity
Source: PLoS One. 2014 Sep 5;9(9):e106796. doi: 10.1371/journal.pone.0106796 (PMC4156407; doi:10.1371/journal.pone.0106796)

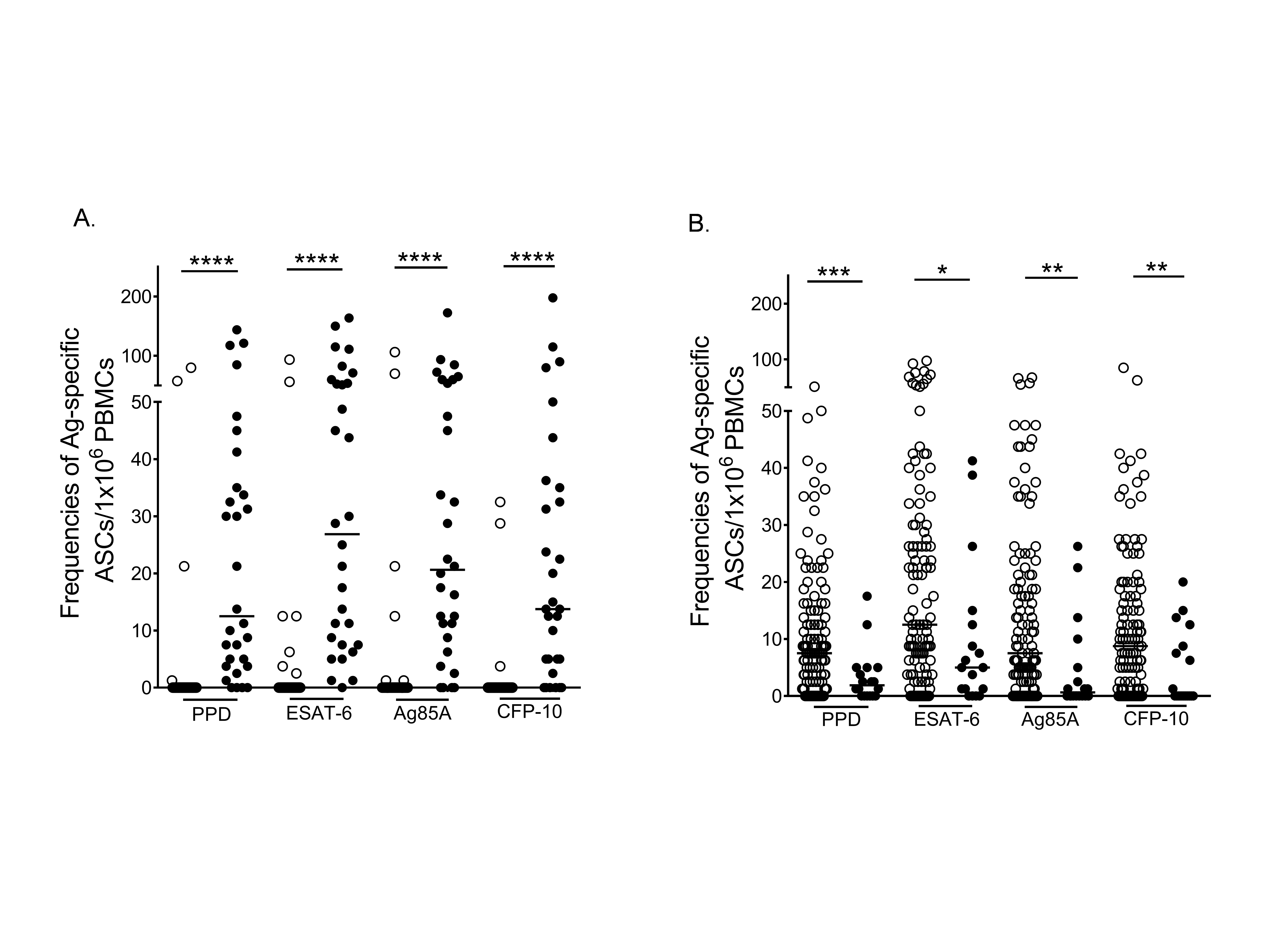

Supplement: Figure S1 — Mycobacteria-specific PB and MBC responses in the healthy community controls and active TB cases. Frequencies of antigen-specific PBs and MBCs were determined by 6-hour ex-vivo and 6-day in-vitro ELISPOTs respectively. Frequencies of antigen-specific PBs and MBCs are presented as antibody secreting cells per million PBMCs. Frequencies of mycobacteria-specific PBs in healthy community controls (open circles) and active TB patients (closed circles) (A) and MBCs in healthy community controls (open circles) and active TB patients (closed circles) (B) are shown. ****, P<0.0001 ***, P<0.001; **, P<0.01, *, P<0.05. (TIF) [file pone.0106796.s001.tif]
